# Supplementary material for: Author-level data confirm the widening gender gap in publishing rates during COVID-19
Source: eLife. 2022 Mar 16;11:e76559. doi: 10.7554/eLife.76559 (PMC8942470; doi:10.7554/eLife.76559)
Supplement: Figure 2—source data 2. [file elife-76559-fig2-data2.docx]

**Figure 2-source data 2.** OLS linear regression with fractional count as dependent variable. Linear regression with author and year fixed effects. Standard errors are HC1 and clustered at the author level.

|  | **Coef.** | **S.E.** | **t-value** | ***Pr(T ≥\|t\|)*** |
| --- | --- | --- | --- | --- |
| Gender x 2016 | 0.0107 | 0.0011 | 10.113 | 0.0000 |
| Gender x 2017 | 0.0036 | 0.0010 | 3.4956 | 0.0005 |
| Gender x 2018 | 0.0017 | 0.0010 | 1.6455 | 0.0999 |
| Gender x 2019 | Ref. | Ref. | Ref. | Ref. |
| Gender x 2020 | -0.0106 | 0.0010 | -10.734 | 0.0000 |
| Num. obs. | 2,041,260 |  |  |  |
| Num. clusters | 408,252 |  |  |  |
| RMSE | 0.211398 |  |  |  |
| Adj *R^2^* | 0.476962 |  |  |  |
| Within *R^2^* | 0.0003 |  |  |  |
