# Supplementary material for: Author-level data confirm the widening gender gap in publishing rates during COVID-19
Source: eLife. 2022 Mar 16;11:e76559. doi: 10.7554/eLife.76559 (PMC8942470; doi:10.7554/eLife.76559)
Supplement: Figure 2—source data 3. [file elife-76559-fig2-data3.docx]

**Figure 2-source data 3.** Poisson regression with full count as dependent variable. Generalized linear regression with author and year fixed effects. Standard errors are HC1 and clustered at the author level.

|  | **Coef.** | **S.E.** | **t-value** | ***Pr(T ≥\|t\|)*** |
| --- | --- | --- | --- | --- |
| Gender x 2016 | -0.0526 | 0.0056 | -9.4591 | 0.0000 |
| Gender x 2017 | -0.0262 | 0.0045 | -5.8706 | 0.0000 |
| Gender x 2018 | -0.0026 | 0.0041 | -0.6157 | 0.5381 |
| Gender x 2019 | Ref. | Ref. | Ref. | Ref. |
| Gender x 2020 | -0.0954 | 0.0044 | -21.753 | 0.0000 |
| Num. obs. | 2,041,260 |  |  |  |
| Num. clusters | 408,252 |  |  |  |
| Adj Pseudo *R^2^* | 0.1957 |  |  |  |
| BIC | 10828121.8 |  |  |  |
