# Supplementary material for: Author-level data confirm the widening gender gap in publishing rates during COVID-19
Source: eLife. 2022 Mar 16;11:e76559. doi: 10.7554/eLife.76559 (PMC8942470; doi:10.7554/eLife.76559)
Supplement: Figure 3—source data 1. [file elife-76559-fig3-data1.docx]

**Figure 3-source data 1.** OLS linear regression of the early-career sample, with full count as dependent variable. Linear regression with author and year fixed effects. Standard errors are HC1 and clustered at the author level.

|  | **Coef.** | **S.E.** | **t-value** | ***Pr(T ≥\|t\|)*** |
| --- | --- | --- | --- | --- |
| Gender x 2016 | 0.0841 | 0.0062 | 13.495 | 0.000 |
| Gender x 2017 | 0.0212 | 0.0062 | 3.4476 | 0.006 |
| Gender x 2018 | -0.0006 | 0.0063 | -0.1016 | 0.9191 |
| Gender x 2019 | Ref. | Ref. | Ref. | Ref. |
| Gender x 2020 | -0.063 | 0.0061 | -10.395 | 0.0000 |
| Num. obs. | 1,391,825 |  |  |  |
| Num. clusters | 278,365 |  |  |  |
| RMSE | 1,0374 |  |  |  |
| Adj. *R^2^* | 0,3046 |  |  |  |
| Within *R^2^* | 0,0005 |  |  |  |
