# Supplementary material for: Author-level data confirm the widening gender gap in publishing rates during COVID-19
Source: eLife. 2022 Mar 16;11:e76559. doi: 10.7554/eLife.76559 (PMC8942470; doi:10.7554/eLife.76559)
Supplement: Figure 3—source data 2. [file elife-76559-fig3-data2.docx]

**Figure 3-source data 2.** OLS linear regression of the mid-career sample, with full count as dependent variable. Linear regression with author and year fixed effects. Standard errors are HC1 and clustered at the author level.

|  | **Coef.** | **S.E.** | **t-value** | ***Pr(T ≥\|t\|)*** |
| --- | --- | --- | --- | --- |
| Gender x 2016 | 0.0635 | 0.0133 | 4.775 | 0.0000 |
| Gender x 2017 | 0.0106 | 0.0124 | 0.8478 | 0.3965 |
| Gender x 2018 | 0.0171 | 0.0119 | 1.4325 | 0.152 |
| Gender x 2019 | Ref. | Ref. | Ref. | Ref. |
| Gender x 2020 | -0.1424 | 0.0123 | -11.613 | 0.0000 |
| Num. obs. | 649,435 |  |  |  |
| Num. clusters | 129,887 |  |  |  |
| RMSE | 1.4641 |  |  |  |
| Adj. *R^2^* | 0,6116 |  |  |  |
| Within *R^2^* | 0,0006 |  |  |  |
