# Supplementary material for: Author-level data confirm the widening gender gap in publishing rates during COVID-19
Source: eLife. 2022 Mar 16;11:e76559. doi: 10.7554/eLife.76559 (PMC8942470; doi:10.7554/eLife.76559)
Supplement: Figure 3—source data 3. [file elife-76559-fig3-data3.docx]

**Figure 3-source data 3.** OLS linear regression of the early-career sample, with fractional count as dependent variable. Linear regression with author and year fixed effects. Standard errors are HC1 and clustered at the author level.

|  | **Coef.** | **S.E.** | **t-value** | ***Pr(T ≥\|t\|)*** |
| --- | --- | --- | --- | --- |
| Gender x 2016 | 0.0166 | 0.0011 | 15.334 | 0.0000 |
| Gender x 2017 | 0.0047 | 0.0011 | 4.3668 | 0.0000 |
| Gender x 2018 | 0.0009 | 0.0011 | 0.7666 | 0.4433 |
| Gender x 2019 | Ref. | Ref. | Ref. | Ref. |
| Gender x 2020 | -0.007 | 0.0011 | -6.6833 | 0.0000 |
| Num. obs. | 1,391,825 |  |  |  |
| Num. clusters | 278,365 |  |  |  |
| RMSE | 0,1832 |  |  |  |
| Adj. *R^2^* | 0,2938 |  |  |  |
| Within *R^2^* | 0,0005 |  |  |  |
