# Supplementary material for: Author-level data confirm the widening gender gap in publishing rates during COVID-19
Source: eLife. 2022 Mar 16;11:e76559. doi: 10.7554/eLife.76559 (PMC8942470; doi:10.7554/eLife.76559)
Supplement: Figure 3—source data 4. [file elife-76559-fig3-data4.docx]

**Figure 3-source data 3.** OLS linear regression of the mid-career sample, with fractional count as dependent variable. Linear regression with author and year fixed effects. Standard errors are HC1 and clustered at the author level.

|  | **Coef.** | **S.E.** | **t-value** | ***Pr(T ≥\|t\|)*** |
| --- | --- | --- | --- | --- |
| Gender x 2016 | 0.0050 | 0.0024 | 2.139 | 0.0324 |
| Gender x 2017 | 0.0004 | 0.0022 | 0.1623 | 0.871 |
| Gender x 2018 | 0.0036 | 0.0021 | 1.6668 | 0.0955 |
| Gender x 2019 | Ref. | Ref. | Ref. | Ref. |
| Gender x 2020 | -0.0154 | 0.0021 | -7.2568 | 0.0000 |
| Num. obs. | 649,435 |  |  |  |
| Num. clusters | 129,887 |  |  |  |
| RMSE | 0.261 |  |  |  |
| Adj. *R^2^* | 0.5675 |  |  |  |
| Within *R^2^* | 0.0002 |  |  |  |
