# Supplementary material for: Author-level data confirm the widening gender gap in publishing rates during COVID-19
Source: eLife. 2022 Mar 16;11:e76559. doi: 10.7554/eLife.76559 (PMC8942470; doi:10.7554/eLife.76559)
Supplement: Figure 5—source data 1. [file elife-76559-fig5-data1.docx]

**Figure 5-source data 1.** OLS linear regression of counterfactual sample, with full count as dependent variable. OLS linear regression of counterfactual sample, with full count as dependent variable.

|  | **Coef.** | **S.E.** | **t-value** | ***Pr(T ≥\|t\|)*** |
| --- | --- | --- | --- | --- |
| Gender x 2011 | 0.1354 | 0.0069 | 19.564 | 0.0000 |
| Gender x 2012 | 0.0698 | 0.0068 | 10.2 | 0.0000 |
| Gender x 2013 | 0.0301 | 0.0069 | 4.3362 | 0.0000 |
| Gender x 2014 | Ref. | Ref. | Ref. | Ref. |
| Gender x 2015 | -0.0501 | 0.0064 | -7.8591 | 0.0000 |
| Num. obs. | 1,132,210 |  |  |  |
| Num. clusters | 226,442 |  |  |  |
| RMSE | 1.019 |  |  |  |
| Adj. *R^2^* | 0.2758 |  |  |  |
| Within *R^2^* | 0.0009 |  |  |  |
