# Supplementary material for: Author-level data confirm the widening gender gap in publishing rates during COVID-19
Source: eLife. 2022 Mar 16;11:e76559. doi: 10.7554/eLife.76559 (PMC8942470; doi:10.7554/eLife.76559)
Supplement: Figure 5—source data 2. [file elife-76559-fig5-data2.docx]

**Figure 5-source data 2.** OLS linear regression of counterfactual sample, with fractional count as dependent variable. Linear regression with author and year fixed effects. Standard errors are HC1 and clustered at the author level.

|  | **Coef.** | **S.E.** | **t-value** | ***Pr(T ≥\|t\|)*** |
| --- | --- | --- | --- | --- |
| Gender x 2011 | 0.0270 | 0.0014 | 19.401 | 0.0000 |
| Gender x 2012 | 0.0118 | 0.0014 | 8.3993 | 0.0000 |
| Gender x 2013 | 0.0028 | 0.0014 | 1.9905 | 0.0465 |
| Gender x 2014 | Ref. | Ref. | Ref. | Ref. |
| Gender x 2015 | -0.0061 | 0.0013 | -4.755 | 0.0000 |
| Num. obs. | 1,132,210 |  |  |  |
| Num. clusters | 226,442 |  |  |  |
| RMSE | 0.208 |  |  |  |
| Adj. *R^2^* | 0.274 |  |  |  |
| Within *R^2^* | 0.0008 |  |  |  |
