# Supplementary material for: Author-level data confirm the widening gender gap in publishing rates during COVID-19
Source: eLife. 2022 Mar 16;11:e76559. doi: 10.7554/eLife.76559 (PMC8942470; doi:10.7554/eLife.76559)
Supplement: Figure 5—source data 3. [file elife-76559-fig5-data3.docx]

**Figure 5-source data 3.** Coefficients and standard errors relative to 2019 for the 30 countries with most authors in the dataset.

| **Country** | **Coef 2016 (S.E.)** | **Coef 2017 (S.E.)** | **Coef 2018 (S.E.)** | **Coef 2020 (S.E.)** |
| --- | --- | --- | --- | --- |
| Poland | 0.083 (0.051) | 0.099 (0.048) | 0.063 (0.05) | -0.12 (0.053) |
| United Kingdom | 0.035 (0.023) | 0.036 (0.022) | 0.00041 (0.023) | -0.086 (0.022) |
| India | 0.087 (0.031) | 0.057 (0.029) | 0.071 (0.03) | -0.12 (0.029) |
| United States | 0.024 (0.012) | -0.025 (0.012) | -0.026 (0.012) | -0.081 (0.011) |
| Turkey | -0.15 (0.047) | -0.041 (0.041) | 0.023 (0.041) | -0.099 (0.042) |
| Germany | 0.099 (0.024) | 0.06 (0.023) | 0.035 (0.022) | -0.13 (0.022) |
| Sweden | -0.008 (0.051) | -0.022 (0.048) | -0.058 (0.052) | -0.12 (0.052) |
| Iran | 0.22 (0.057) | 0.28 (0.056) | 0.18 (0.05) | -0.25 (0.051) |
| Brazil | 0.11 (0.025) | 0.1 (0.024) | 0.029 (0.026) | -0.11 (0.026) |
| China | 0.15 (0.028) | 0.13 (0.027) | 0.077 (0.026) | -0.073 (0.025) |
| Israel | -0.0037 (0.058) | 0.018 (0.054) | -0.078 (0.056) | -0.12 (0.059) |
| Russia | 0.13 (0.047) | 0.13 (0.045) | 0.1 (0.047) | -0.16 (0.049) |
| Japan | 0.11 (0.023) | 0.076 (0.023) | 0.053 (0.024) | -0.11 (0.022) |
| Argentina | 0.021 (0.05) | 0.025 (0.051) | -0.022 (0.057) | -0.084 (0.055) |
| France | 0.086 (0.028) | 0.045 (0.027) | 0.026 (0.028) | -0.11 (0.028) |
| Italy | 0.17 (0.034) | 0.13 (0.031) | 0.1 (0.03) | -0.19 (0.035) |
| Portugal | 0.019 (0.068) | 0.014 (0.068) | -0.079 (0.067) | -0.1 (0.073) |
| Mexico | 0.15 (0.049) | 0.079 (0.043) | 0.053 (0.048) | -0.098 (0.046) |
| Canada | -0.015 (0.033) | -0.02 (0.031) | -0.0083 (0.031) | -0.083 (0.03) |
| Switzerland | 0.012 (0.049) | 0.041 (0.047) | -0.014 (0.047) | -0.11 (0.046) |
| Netherlands | 0.027 (0.048) | 0.018 (0.045) | 0.053 (0.045) | -0.091 (0.044) |
| Austria | 0.15 (0.071) | 0.054 (0.067) | 0.087 (0.068) | -0.22 (0.07) |
| Australia | 0.067 (0.038) | -0.0038 (0.037) | 0.028 (0.038) | -0.044 (0.037) |
| South Korea | 0.089 (0.055) | 0.14 (0.054) | 0.093 (0.05) | -0.14 (0.046) |
| Spain | 0.038 (0.031) | 0.003 (0.029) | -0.016 (0.03) | -0.11 (0.03) |
| Egypt | 0.17 (0.061) | 0.14 (0.06) | -0.0077 (0.061) | -0.085 (0.073) |
| Denmark | 0.021 (0.064) | 0.076 (0.061) | 0.022 (0.06) | -0.015 (0.058) |
| Belgium | 0.011 (0.06) | 0.04 (0.057) | -0.071 (0.058) | -0.045 (0.059) |
| Pakistan | 0.22 (0.089) | 0.25 (0.084) | 0.15 (0.079) | -0.045 (0.077) |
| Czech Republic | 0.16 (0.07) | 0.17 (0.068) | 0.14 (0.07) | -0.083 (0.069) |
