# Supplementary material for: Author-level data confirm the widening gender gap in publishing rates during COVID-19
Source: eLife. 2022 Mar 16;11:e76559. doi: 10.7554/eLife.76559 (PMC8942470; doi:10.7554/eLife.76559)
Supplement: Figure 6—source data 1. [file elife-76559-fig6-data1.docx]

**Figure 6-source data 1.** Coefficients and standard errors relative to 2019 for the four disciplines.

| **Country** | **Coef 2016 (S.E.)** | **Coef 2017 (S.E.)** | **Coef 2018 (S.E.)** | **Coef 2020 (S.E.)** |
| --- | --- | --- | --- | --- |
| Clinical medicine | 0.045 (0.0086) | 0.0017 (0.0083) | -0.0028 (0.0083) | -0.12 (0.0083) |
| Chemistry | 0.11 (0.017) | 0.086 (0.016) | 0.044 (0.016) | -0.062 (0.015) |
| Biology | 0.078 (0.011) | 0.051 (0.01) | 0.04 (0.011) | -0.09 (0.011) |
| Basic medicine | 0.06 (0.015) | 0.033 (0.014) | -0.0079 (0.014) | -0.058 (0.014) |
