# Supplementary material for: Author-level data confirm the widening gender gap in publishing rates during COVID-19
Source: eLife. 2022 Mar 16;11:e76559. doi: 10.7554/eLife.76559 (PMC8942470; doi:10.7554/eLife.76559)
Supplement: Figure 7—source data 2. — Linear regression with author and year fixed effects. Standard errors in parentheses are HC1 and clustered at the author level. [file elife-76559-fig7-data2.docx]

**Figure 7-source data 2.** OLS linear regression of full and fractional count as dependent variable, placebo test of 2018 vs. 2019. Linear regression with author and year fixed effects. Standard errors in parentheses are HC1 and clustered at the author level.

|  | **Full count** |  | **Fractional count** |  |
| --- | --- | --- | --- | --- |
|  | **Coef. (S.E.)** | ***Pr(T≥\|t\|)*** | **Coef. (S.E.)** | ***Pr(T≥\|t\|)*** |
| Gender × 2016 | 0.0588 | 0.0000 | 0.0089 | 0.0000 |
|  | (0.0052) |  | (0.0010) |  |
| Gender × 2017 | 0.0145 | 0.0025 | 0.0019 | 0.0313 |
|  | (0.0048) |  | (0.0009) |  |
| Gender × 2018 | Ref. | Ref. | Ref. | Ref. |
|  |  |  |  |  |
| Gender × 2019 | -0.0095 | 0.0893 | -0.0023 | 0.0209 |
|  | (0.0056) |  | (0.0010) |  |
| Num. obs. | 1724828 | & 1724828 |  |  |
| Num. clusters | 431207 | & 431207 |  |  |
| RMSE | 1.0719 | & 0.1960 |  |  |
| Adj. *R^2^* | 0.5160 | & 0.4752 |  |  |
| Within *R^2^* | 0.0001 | & 0.0001 |  |  |
| Gender × 2016 | 0.0588 | 0.0000 | 0.0089 | 0.0000 |
|  | (0.0052) |  | (0.0010) |  |
